# 

**Published:** 1840

**Authors:** 


### CONTENTS

OF NO'S. II, III, IV, AND V.

### ORIGINAL COMMUNICATIONS.

### ESSAYS.

ART. I.—An Essay on the Summer and Autumnal Remittent Fevers of Mississippi. By JOHN W. MONETTE, M. D. 87

ART. II.—Surgical and Pathological Observations on the Eye. By WILLIAM A. MCDOWELL, M. D., - - - - 130

ART. III.—An Inquiry into Dysenteric Bilious Fever. By W. K. BEWLING, M. D. - - - - - - - 166

ART. IV.—Facts relative to Milk-Sickness. By WM. J. BARBEE, M. D. - - - - - - - - - 178

ART. V.—Practical observations on the same. By DR. M. WINANS. - - - - - - - - - 191

ART. VI.—History of a Case of Intussusceptio. By DR. JOHN DAWSON. - - - - - - - - - 193

ART. VII.—An account of a case of Tubercular Disease. By GEO. W. BAYLESS, M. D. - - - - - - - 196

ART. VIII.—A Case of Extra-Uterine Pregnancy. By DR. SAMUEL SEXTON. - - - - - - - - 207

ART. IX.—The Causes and Treatment of Convulsive Affections of Children. By ALFRED MILLIKIN, M. D. - - 209

ART. X.—Partial Deafness and Blindness occurring in a Case of Pneumonitis. By DR. T. J. COGLEY. - - - 215
ART. XI.—Observations on the use of large doses of Acetate of Lead in Fever. By A. KIMBALL, M. D. - - 218
ART. XII. A Case of Puerperal Convulsions. By JOHN M. BIGELOW, M. D. - - - - - - - - 224
ART. XIII.—A Case of Tænia Solium causing Herpes. By the Same. - - - - - - - - - 229
ART. XIV.—A Note on the supposed cause of Milk-Sickness. By CHARLES W. SHORT, M. D. - - - - - 231

REVIEWS.

ART. XV.—Lectures on the Theory and Practice of Physic. By DAVID HOSACK, M. D. - - - - - - 233
ART. XVI.—The Medical Examiner, and American Medical Library; a reply to their Strictures. - - - - - 256
ART.—XVII.—Analysis of an Article in the American Journal of the Medical Sciences, on Hanging. - - - 264

BIBLOGRAPHICAL NOTICES.

ART. XVIII.—Statistics of the Medical Colleges of the U. S. By T. ROMEYN BECK, M. D. - - - - - - 273
ART. XIX.—A Catalogue of Plants in the vicinity of Columbus, Ohio. By WM. S. SULLIVANT. - - - - 278
ART. XX – The Philadelphia Practice of Midwifery. By CHARLES D. MEIGS, M. D. - - - - - - - - 280

SELECTIONS FROM AMERICAN AND FOREIGN JOURNALS.

A Monograph on Excisions. By MALGAIGNE. - - - - - 293
Excisions of Enlarged Tonsils. - - - - - - - - - 317
Nitro-Muriatic acid as a remedial agent. - - - - - - 317

Remarks on a Person who had been Beheaded. - - - - 319
Poisoning with Arsenic—Late Discoveries. - - - - - 328
Sir B. Brodic on Calculus. - - - - - - - - - 335
Treatment of Paralysis. - - - - - - - - - - 336
Case of a Child with two heads. - - - - - - - - 336
Case of Ozœna and Neuralgia, cured by the extraction of a Tooth. - 337
Case of Stone in a Girl relieved by Lithontritie. - - - - 339
Mortality in New York in 1839. - - - - - - - - 339
Internal Uterine Hemorrhage. - - - - - - - - 341
Esquirol on Insanity. - - - - - - - - - - 350
The Pellicle of an Egg, an adhesive application to wounds. - - 352
Saratoga Springs. - - - - - - - - - - - 353
Dialogue or Mechanism *versus* Vitalism. - - - - - - 354
On Lightning Rods and their improvement. By Dr. Hare. - - 355

DOMESTIC INTELLIGENCE.

Our Delay. - - - - - - - - - - - - 359
Editorial Department. - - - - - - - - - - 360
Variations in Quantity of Rain—Malaria, - - - - - 360
Death from Salivation in a Child. - - - - - - - 361
Commercial Hospitals in the West. - - - - - - 362
Proposed Remedy for Spina Bifida. - - - - - - 362
Production of Castor oil in Illinois. - - - - - - 364
Pathological Museum of the Medical Institute, L. - - - 365
Convention of the Physicians of N. E. Kentucky. - - - - 365
National Medical Convention in Philadelphia. - - - - 366
Case of Triplets. - - - - - - - - - - - 368
Case of Anasarca. - - - - - - - - - - 368
Monstrosity. - - - - - - - - - - - - 369
Baltimore College of Dental Surgery. - - - - - - 369
Clarendon Springs. - - - - - - - - - - 370
Winter Retreat for the Consumptive. - - - - - - 370
Medical Society of Stark Co. Ohio. - - - - - - 371
Cure of Congenital Deafness. - - - - - - - 372

Asylums for the Deaf and Dumb. - - - - - - - 373
Egyptian School of Medicine. - - - - - - - 375
Espy's Lectures on Meteorology. - - - - - - - 377
Thunderstorm. - - - - - - - - - - 378
Combe's Lectures on Phrenology. - - - - - - - 379
Harrodsburg Springs. - - - - - - - - - 379
Subterranean Retreat for Invalids. - - - - - - 379
Lunatic Asylums. - - - - - - - - - 380
Candidates for Admission into the Army. - - - - - 384
Death of *Dr. Parrish.* - - - - - - - - 387
Animal Magnetism. - - - - - - - - - 388
Hospitals of London, Paris, Dublin and Edinburg. - - - 388
Paroquet Springs. - - - - - - - - - 395
Medical Society of Tennessee. - - - - - - - 297
Medical Institute of Louisville—Professor of Surgery. - - 399

